# The Optimal Time of Ovarian Reserve Recovery After Laparoscopic Unilateral Ovarian Non-Endometriotic Cystectomy

**DOI:** 10.3389/fendo.2021.671225

**Published:** 2021-09-24

**Authors:** Huaping Li, Bin Yan, Yanli Wang, Zhiming Shu, Ping Li, Yahong Liu, Ying Wang, Xiaohong Ni, Zhou Liu

**Affiliations:** ^1^ Department of Obstetrics and Gynecology, Shanghai University of Medicine & Health Sciences Affiliated Zhoupu Hospital, Shanghai, China; ^2^ Department of Obstetrics and Gynecology, Shanghai Punan Hospital, Shanghai, China; ^3^ Department of Obstetrics and Gynecology, Ren Ji Hospital School of Medicine, Shanghai Jiao Tong University, Shanghai, China; ^4^ Department of Obstetrics and Gynecology, The First People’s Hospital of Zhengzhou, Zhengzhou, China; ^5^ Shanghai University of Medicine & Health Sciences, Shanghai, China

**Keywords:** ovarian reserve, unilateral ovarian non-endometriotic cyst, laparoscopic cystectomy, fertility, optimal time

## Abstract

**Background:**

Laparoscopic ovarian cystectomy is established as the standard surgical approach for the treatment of benign ovarian cysts. However, previous studies have shown that potential fertility can be directly impaired by laparoscopic ovarian cystectomy, diminished ovarian reserve (DOR), and even premature ovarian failure. Therefore, fertility-preserving interventions are required for benign gynecologic diseases. However, there are still little data on the time period required for recovery of ovarian reserve after the laparoscopic unilateral ovarian cystectomy, which is very important for the individualization of treatment protocols. This study aimed at investigating the time needed for the ovarian reserve to recover after laparoscopic unilateral ovarian non-endometriotic cystectomy.

**Materials and Methods:**

Sixty-seven patients with unilateral ovarian non-endometriotic cyst from Zhoupu and Punan Hospitals who underwent laparoscopic unilateral ovarian cystectomy were recruited as a postoperative observation group (POG). Also, 69 healthy age-matched women without ovarian cyst who did not undergo surgery were recruited as a referent group (RFG). Ovarian reserve with the serum anti-Müllerian hormone (AMH), follicle-stimulating hormone (FSH), estradiol (E2) levels, ovarian arterial resistance index (OARI), and antral follicle counts (AFCs) were measured on the third to fifth days of the same menstrual cycle. A postoperative 6-month follow-up of cases was performed.

**Results:**

Compared with RFG, AFC of cyst side in the POG group showed no difference in the first, third, and sixth postoperative month (F = 0.03, F = 0.02, F = 0.55, respectively; p = 0.873, p = 0.878, p = 0.460, respectively). The OARI of cyst side in the POG group revealed no differences in the first, third, and sixth postoperative month (F = 0.73, F = 3.57, F = 1.75, respectively; p = 0.395, p = 0.061, p = 0.701, respectively). In the first month, the postoperative AMH levels significantly declined, reaching 1.88 ng/ml [interquartile range (IQR): 1.61–2.16 ng/ml] in POG and 2.57 ng/ml (IQR: 2.32–2.83 ng/ml) in RFG (F = 13.43, p = 0.000). For the data of AMH levels stratified by age, the same trend was observed between less than 25 and more than 26 years old. At this same time interval, the postoperative rate of decline was significantly lower compared to the preoperative one in POG (32.75%). The same trend was observed between the POG and RFG groups (26.67%).

**Conclusions:**

The optimal time for recovery of ovarian reserve after laparoscopic unilateral ovarian cystectomy is estimated to be 6 months.

## Introduction

Benign ovarian cysts, frequently found in women of reproductive age, are among the most important detrimental causes affecting ovarian reserve (1, 2). Laparoscopic ovarian cystectomy is established as the standard surgical approach for the treatment of benign ovarian cysts (3). Ovarian reserve is defined as a woman’s reproductive potential in terms of the number and quality of her remaining oocytes (2). However, previous studies have shown that potential fertility can be directly impaired by laparoscopic ovarian cystectomy (4–6), diminished ovarian reserve (DOR), and even premature ovarian failure (7–9). Many previous studies have confirmed the ovarian reserve damage after laparoscopic stripping of endometrioma (10–15). Benign gynecological diseases are often implicated in infertility problems. Therefore, fertility-preserving interventions are required for benign gynecologic diseases (12).

Anti-Müllerian hormone (AMH) level testing is a useful screening test for assessing ovarian reserve in women at high risk of diminished ovarian reserve (16), especially for young women with cancer (17). According to a previous study, there was no significant decrease in the serum of AMH levels 3 months after endometrioma cystectomy (18). In contrast, the recovery of AMH serum level was observed from 3 to 6 months after endometrioma cystectomy (19). However, there are still little data on the time period required for recovery of ovarian reserve after the laparoscopic unilateral ovarian cystectomy, which is very important for the individualization of treatment protocols. Consequently, the aim of this study was to investigate the time needed for recovery of ovarian reserve after laparoscopic unilateral ovarian non-endometriotic cystectomy.

## Materials and Methods

### Study Design and Participants

This prospective cohort study was conducted in the Gynecology Department of Shanghai University of Medicine & Health Sciences Affiliated Zhoupu Hospital and Punan Hospital between 2016 and 2019. A total of 67 patients with unilateral ovarian benign non-endometriotic cyst who underwent a laparoscopic cystectomy were invited to participate in the postoperative observation group (POG). Serum samples were continuously collected from these prospectively enrolled women to be used to ascertain the time needed for recovery of ovarian reserve after the laparoscopic unilateral ovarian cystectomy. The inclusion criteria were as follows: 1) age 20–30 years; 2) regular menstrual bleeding (cycle length: between 21 and 45 days); 3) diameter of unilateral ovarian cysts: 4–10 cm; 4) normal preoperative level of serum tumor markers; 5) benign ovarian tumor of intraoperative rapid frozen pathology; 6) not pregnant and not planning on getting pregnant in the following 6 months. Exclusion criteria were the following: 1) any suspicious finding of malignant ovarian diseases; 2) ovarian, endometrial cyst; 3) ovarian, uterine, tubal surgery history; 4) endocrine disease and treatment history; 5) severe medical or surgical complications; 6) smoking.

Sixty-nine age-matched women were enrolled as the referent group (RFG). The inclusion criteria were following: 1) age 20–30 years old; 2) regular menstrual bleeding (cycle length: between 21 and 45 days); 3) normal preoperative level of serum tumor markers; 4) not pregnant and not planning on getting pregnant within the next 6 months. Exclusion criteria were the following: 1) endocrine disease and treatment history; 2) ovarian, uterine, and tubal surgery history; 3) severe medical or surgical complications; 4) smoking. The current study was approved by the Committee of Medical Ethics, Shanghai Punan Hospital (No. 2014031). All the women provided written informed consent before participation.

### Laparoscopic Ovarian Cystectomy

The laparoscopic unilateral ovarian cystectomy was performed under general anesthesia by the same surgical team with 10 years of laparoscopic experience. The weak position of the cyst surface was opened with an ultrasound knife upon the visual exploration of the pelvic cavity and ovarian cysts, after which the cyst was completely detached from the ovarian cortex while saving the healthy ovarian cortex as much as possible. During the operation, hemostasis was achieved with bipolar electrocoagulation forceps at the power of 25 W and for the duration of no more than 5 s. A loose knot was made of 2/0 absorbable sutures for controlling bleeding and reshaping ovarian morphology. The specimens were examined under intraoperative rapid freezing pathology in order to exclude malignancy, which was followed by the routine pathological examination.

After the surgery, the women were monitored and observed in the hospital wards for 48 h for surgical or anesthesia-associated complications. For all the women, the operative and postoperative courses were successful without any specific complications.

### Measurements of Postoperative Pormone and Ultrasonic Examination Items

In POG, the fasting blood of each woman was collected on the morning of the second day of the menstrual cycle to be examined 1 month prior to the laparoscopic unilateral ovarian cystectomy; the same collecting procedure was performed in the RFG group. The serum was separated from the whole blood and transferred into a sterile polypropylene tube to be stored at -80°C. After the operation, the samples were examined in the first, third, and sixth month.

In RFG, the serum was collected at the same time point. The serum AMH levels were measured using a commercially available enzyme-linked immunosorbent assay kit (Beckman, Germany); the follicle-stimulating hormone (FSH) and estradiol (E2) levels were measured using a chemiluminescent reagent kit (Siemens, Germany). According to the experimental methods of Rosendahl et al. (20) and Bentzen et al. (21), ovarian arterial resistance index (OARI) and antral follicle count (AFC) were measured by transvaginal ultrasonography (Philips, Germany) on the third to fifth days of the same menstrual cycle.

### Follow-Up and Statistical Analysis

The follow-up was performed from May 2016 to May 2019 by full-time personnel involved with the project. The follow-up method was a combination of face-to-face interviews and telephone follow-up. The follow-up contents included the general situation of the patient, medication time, menstruation, and eventual pregnancy. The age, body mass index (BMI), and serum AMH, FSH, and E2 levels were recorded. The endpoint of the study was to obtain the observation data during the follow-up.

SPSS10.0 software package (SPSS Inc., Chicago, IL, USA) was used for the statistical analysis. For quantitative variables, after the normality of the data was checked, mean ± SD and median (range) were used to describe the normal and non-normal distribution, respectively. One-way ANOVA was used to perform group comparisons. A p-value <0.05 was considered to be statistically significant.

## Results

**Figure 1:**
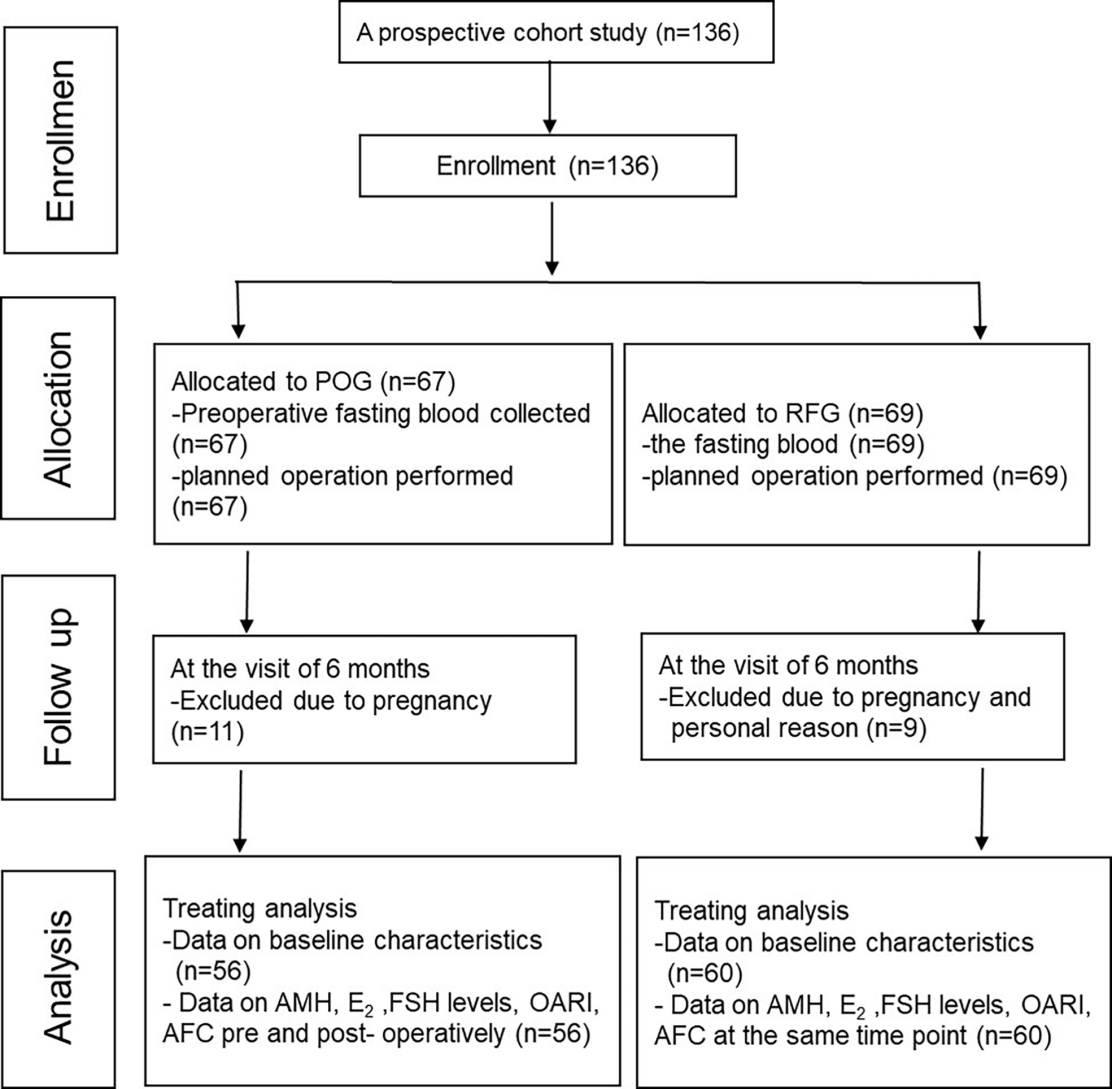
Enrollment, randomization, and follow-up of the study subjects.

This prospective cohort study included 136 patients. Among these, 11 participants were excluded from the POG group, while nine participants from RFG withdrew due to pregnancies and other personal reasons (**Figure 1**).

### Baseline Characteristics

There were no significant differences in age (25.71 ± 3.17 *vs*. 26.35 ± 4.33 years, p = 0.992), BMI (22.51 ± 2.63 *vs*. 23.24 ± 2.78, p = 0.256), OARI (the cyst-side ovary; 0.79 *vs*. 0.75, p = 0.379), AFC (the cyst-side ovary; 6.73 *vs*. 6.76, p = 0.931), and median baseline levels of AMH (2.81 *vs*. 2.61 ng/ml, p = 0.352), E2 (44.43 *vs*. 44.85 ng/ml, p = 0.851), FSH (4.71 *vs*. 4.74 IU/ml, p = 0.930), and CA125 (21.43 *vs*. 18.3 IU/ml, p = 0.324) between POG and RFG groups. Such data were detected 1 month prior to the laparoscopic unilateral ovarian cystectomy (p > 0.05; **Table 1**).

**Table 1:** Baseline characteristics.

Characteristic	POG (n = 56)	RFG (n = 60)	p-value*
Age enrollment (years)	25.71 ± 3.17	26.35 ± 4.33	0.992
BMI (kg/m^2^)	22.51 ± 2.63	23.24 ± 2.78	0.256
Preop CA125 (IU/ml)	21.43 (6.2–25.9)	18.3 (4.3–23.3)	0.324
OARI	0.79 (0.72–0.85)	0.75 (0.70–0.80)	0.379
AFC (n)	6.73 (6.12–7.34)	6.76 (6.26–7.26)	0.931
Preop AMH (ng/ml)	2.81 (2.48–3.12)	2.61 (2.32–2.89)	0.352
Preop E2 (pg/ml)	44.43 (35.28–53.42)	44.85 (35.69–53.12)	0.851
Preop FSH (IU/ml)	4.71 (4.13–5.24)	4.74 (4.44–5.01)	0.930

*As indicated by one-way ANOVA; AMH, anti-Müllerian hormone; E2, estradiol; FSH, follicle-stimulating hormone; POG represents a postoperative observation group that underwent laparoscopic unilateral ovarian cystectomy; RFG represents a referent group that did not undergo surgery; OARI, ovarian arterial resistance index; AFC, antral follicle count; Preop, preoperatively; () The value is 95% confidence interval.

In POG, the cyst size was 4.67 ± 3.12 cm; pathological types were teratoma (13/59 cases, 23.2%), ovarian serous cystadenoma (10/59, 17.9%), ovarian mucinous cystadenoma (9/59, 16.1%), a simple ovarian cyst (11/59, 19.6%), and others (13/59, 23.2%). The indications for surgery were abdominal pain (13/59, 26.7%), risk of torsion (10/59, 17.9%), infertility (9/59, 16.1%), potentially malignant (11/59, 19.6%), and others (13 cases, 23.2%). The duration of surgery was 56.5 ± 22.3 min; blood loss was 50.4 ± 21.6 ml; hospital stay was 3.6 ± 1.4 (days) (**Table 2**).

**Table 2:** Baseline clinical characteristics of POG.

Characteristic	POG (n = 56)
**The diameter of the cyst (cm)**	4.67 ± 3.12
**Pathological type**	
Teratoma	13 (23.2%)
Ovarian serous cystadenoma	10 (17.9%)
Ovarian mucinous cystadenoma	9 (16.1%)
Ovarian simple cyst	11 (19.6%)
Others	13 (23.2%)
**Indication for surgery**	
Abdominal pain	15 (26.7%)
Risk of torsion	14 (25%)
Infertility	11 (19.6%)
Potentially malignant	8 (14.3%)
Others	8 (14.3%)
**Duration of surgery (min)**	56.5 ± 22.3
**Blood loss (ml)**	50.4 ± 21.6
**Hospital stay (days)**	3.6 ± 1.4

POG represents a postoperative observation group that underwent laparoscopic unilateral ovarian cystectomy.

### Changes of Antral Follicle Count and Ovarian Arterial Resistance Index in the Two Groups

**Figure 2:**
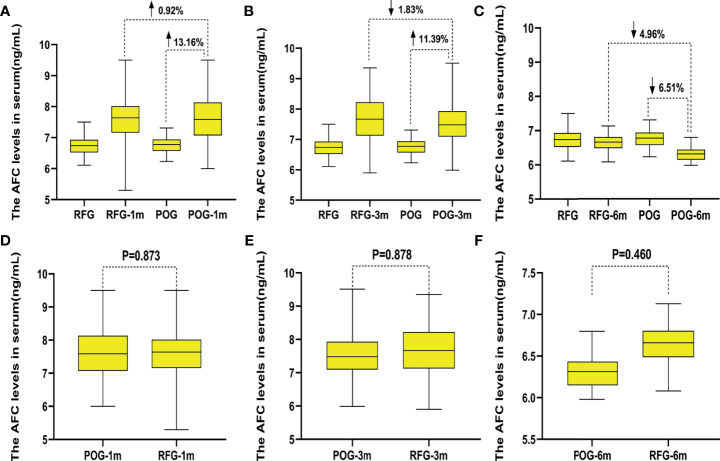
The changes of the antral follicle count (AFC) in the two groups. Compared with preoperative, the increased AFC count of postoperative observation group (POG) in the ovary cyst side after the first postoperative month was 13.16%. Compared with the AFC count of referent group (RFG) in the same ovary side, the increased rate of AFC count of POG in the ovary cyst side after the first postoperative month was 0.92% **(A)**. Compared with preoperative, the increased AFC count of POG in the ovary cyst side after third postoperative month was 11.39%. Compared with the AFC count of RFG in the same ovary side, the declined AFC count of POG in the cyst ovary side after the third postoperative month was 1.83% **(B)**. Compared with preoperative, the declined AFC count of POG in the ovary cyst side after the sixth postoperative month was 6.51%. Compared with the AFC count of RFG in the same ovary side, the increased AFC count of POG in the ovary cyst side after the sixth postoperative month was 4.96% **(C)**. The AFC of the cyst showed no significant difference in POG when compared with that in RFG in the same side ovary in the first, third, and sixth month postoperatively (p = 0.873, p = 0.878, p = 0.460, respectively) **(D–F)**.

**Figure 3:**
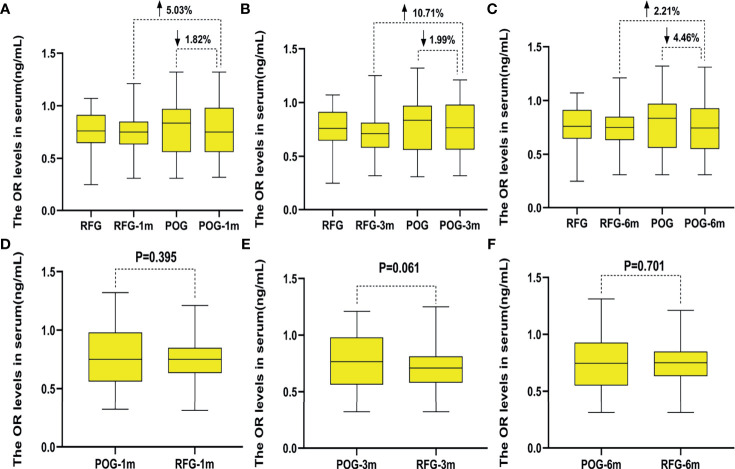
The changes of ovarian arterial resistance index (OARI) in the two groups. Compared with preoperative, the decline rate of OARI in postoperative observation group (POG) of ovary cyst side after the first postoperative month was 1.82%. Compared with the OARI of referent group (RFG) in the same ovary side, the increased rate of OARI in POG of ovary cyst side after the first postoperative month was 5.03% **(A)**. Compared with preoperative, the decline rate of OARI in POG of ovary cyst side after third postoperative month was 1.99%. Compared with the OARI of RFG in the same side ovary, the increased rate of OARI in POG in the ovary cyst side after the third postoperative month was 10.71% **(B)**. Compared with preoperative, the decline rate of OARI in POG of ovary cyst side after the sixth postoperative month was 4.46%. Compared with the OARI of RFG in the ovary cyst side, the increased rate of OARI in POG in the ovary cyst side after the third postoperative month was 2.21% **(C)**. The OARI of the cyst side showed no significant difference in POG when compared with that in RFG in the same side ovary postoperatively in the first, third, and sixth month (p = 0.395, p = 0.061, p = 0.701) **(D–F)**.

The AFC of the cyst side showed no significant difference in POG when compared with RFG in the first, third, and sixth month postoperatively (F = 0.03, F = 0.02, F = 0.55, respectively; p = 0.873, p = 0.878, p = 0.460, respectively; **Table 3** and **Figure 2**). No statistical significances were observed between the three detecting time intervals (F = 0.22, p = 0.808) and between detecting time intervals and grouping (F = 0.32, p = 0.881). In the OARI of the cyst side, no statistical significances were observed between POG and RFG in the first, third, and sixth month postoperatively (F = 0.73, F = 3.57, F = 1.75, respectively; p = 0.395, p = 0.061, p = 0.701, respectively), between three detecting time intervals (F = 1.69, p = 0.185), and between detecting time interval and grouping (F = 1.086, p = 0.355; **Table 3** and **Figure 3**).

**Table 3:** Ovarian reserve by assessment of OARI and AFC preoperatively and postoperatively.

Indicators	POG (n = 56)	RFG (n = 60)	p-value*
**AFC of the cyst side/same side**			
In the first postop. m.	7.58 (6.01–8.31)	7.65 (6.14–8.45)	0.873
In the third postop. m.	7.67 (6.12–8.41)	7.53 (6.05–8.37)	0.878
In the sixth postop. m.	6.65 (6.21–7.53)	6.32 (5.91–7.31)	0.460
**OARI of the cyst side**			
In the first postop. m.	0.78 (0.71–0.95)	0.73 (0.62–0.85)	0.395
In the third postop. m.	0.69 (0.71–0.91)	0.65 (0.65–0.85)	0.061
In the sixth postop. m.	0.75 (0.68–0.92)	0.74 (0.68–0.96)	0.701

*As indicated by ANOVA; POG represents a postoperative observation group that underwent laparoscopic unilateral ovarian cystectomy; RFG represents a referent group that did not undergo surgery; OARI, ovarian arterial resistance index; AFC, antral follicle count; Preop, preoperatively; in the first postop. m, in the first month postoperatively; in the third postop. m., in the third month postoperatively; in the sixth postop. m., in the sixth month postoperatively; (), The value is 25% and 75% of median.

### Changes of Anti-Müllerian Hormone, Estradiol, and Follicle-Stimulating Hormone Levels in the Two Groups

**Figure 4:**
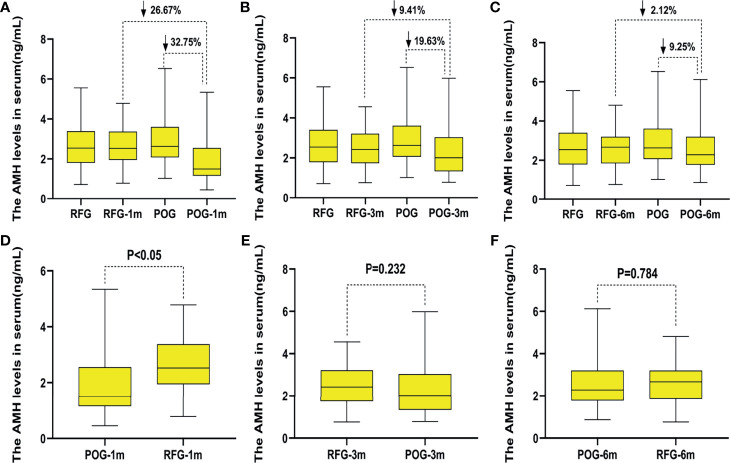
The changes of anti-Müllerian hormone (AMH) levels in the two groups. Compared with preoperative, the decline rate of AMH levels in postoperative observation group (POG) after the first postoperative month was 32.75%. Compared with AMH levels of referent group (RFG), the decline rate of AMH levels in POG after the first postoperative month was 26.67% **(A)**. Compared with preoperative, the decline rate of AMH levels in POG after third postoperative month was 19.63%. Compared with AMH levels of RFG, the declined AMH level of POG after the third postoperative month was 9.41% **(B)**. Compared with preoperative, the decline rate of AMH levels in POG after the sixth postoperative month was 9.25%. Compared with AMH levels of RFG, the decline rate of AMH levels in POG after the third postoperative month was 2.12% **(C)**. Compared with AMH levels of RFG, the AMH levels of POG significantly declined in the first postoperative month {1.88 ng/ml [interquartile range (IQR), 1.61–2.16 ng/ml] *vs*. 2.57 ng/ml (IQR, 2.32–2.83 ng/ml); F = 13.43, p = 0.000} **(D)**. The AMH levels showed no significant difference in POG in the first, third, and sixth month when compared with that in RFG (p = 0.232, p = 0.784, respectively) **(E, F)**.

Interestingly, AMH levels of POG significantly declined in the first postoperative month {1.88 ng/ml [interquartile range (IQR), 1.61–2.16 ng/ml]} when compared with those of RFG [2.57 ng/ml (IQR, 2.32–2.83 ng/ml); F = 13.43, p = 0.000; **Figure 4D**]. At this interval, the decline rate was significantly lower than that preoperatively in POG (32.75%; **Figure 4A**). The same trend was observed when comparing POG with RFG (26.67%; **Figure 4A**). Compared with preoperative, the decline rate of AMH levels in POG after third postoperative month was 19.63%. Compared with AMH levels of RFG, the declined AMH level of POG after the third postoperative month was 9.41% (**Figure 4B**). Compared with preoperative, the decline rate of AMH levels in POG after the sixth postoperative month was 9.25%. Compared with AMH levels of RFG, the decline rate of AMH levels in POG after the third postoperative month was 2.12% (**Figure 4C**). However, in the third and sixth month, the postoperative AMH levels were found to be similar between POG and RFG (F = 1.42, F = 0.75, respectively; p = 0.232, p = 0. 784, respectively) (**Table 4** and **Figures 4E, F** **)**. These results showed that postoperative AMH levels were gradually restored to the preoperative levels in the sixth month, revealing a statistical significance between three detection time intervals (F = 14.21; p = 0.000). As indicated by **Table 4**, the interaction between the detection time and grouping was statistically significant (F = 111.89, p = 0.000).

**Table 4:** Ovarian reserve by assessment of AMH, E2, FSH levels preoperatively and postoperatively.

Indicator	POG (n = 56)	RFG (n = 60)	p-value*
**AMH level (ng/ml)**			
Preop. AMH	2.81 (2.48–3.12)	2.61 (2.32–2.89)	0.453
In the first postop. m.	1.88 (1.61–2.16)^#^	2.57 (2.32–2.83)	0.000
In the third postop. m.	2.26 (2.32–2.74)^#^	2.49 (1.96–2.55)	0.232
In the sixth postop. m.	2.41 (2.24–2.85)	2.60 (2.34–2.87)	0.784
**E2 level (pg/ml)**			
Preop. E2	44.43 (35.28–53.42)	44.85 (35.69–53.12)	0.728
In the first postop. m.	45.16 (31.91–62.15)	46.09 (33.45–68.72)	0.645
In the third postop. m.	43.07 (36.86–69.28)	42.91 (34.67–65.32)	0.638
In the sixth postop. m.	40.80 (33.79–60.13)	47.18 (32.89–71 53)	0.913
**FSH level (IU/ml)**			
Preop FSH (IU/ml)	4.71 (4.13–5.24)	4.74 (4.44–5.01)	0.576
In the first postop. m.	4.32 (3.86–5.03)	4.35 (4.01–5.13)	0.872
In the third postop. m.	4.54 (4.52–5.26)	4.40 (4.05–5.31)	0.878
In the sixth postop. m.	4.35 (3.67–5.16)	4.49 (4.03–5.43)	0.459

*As indicated by one-way ANOVA, ^#^As indicated by one-way ANOVA, the difference was statistically significant when compared with preoperative. AMH, anti-Müllerian hormone; E2, estradiol; FSH, follicle-stimulating hormone; POG, a postoperative observation group that underwent laparoscopic unilateral ovarian cystectomy; RFG, a referent group that did not undergo surgery; OARI, ovarian arterial resistance index; AFC, antral follicle count; Preop, preoperatively; in the first postop. m, in the first month postoperatively; in the third postop. m., in the third month postoperatively; in the sixth postop. m., in the sixth month postoperatively; (), The value is 95% confidence interval.

Considering that AMH peaked at about 25 years of age, we stratified the data by age. We got similar results when the age was less than 25 years old; AMH level of POG in the first postoperative month was significantly lower than that in RFG [1.75 ng/ml (IQR, 1.35–2.15 ng/ml) and 2.78 ng/ml (IQR, 2.37–3.18 ng/ml), p = 0.001] (**Table 5** **)**. When the age was more than 26 years old, AMH level of POG in the first postoperative month was significantly lower than that in RFG [1.53 ng/ml (IQR, 1.32–2.32 ng/ml) and 2.49 ng/ml (IQR, 2.01–2.87 ng/ml), p = 0.041) **(** **Table 6** **).**

**Table 5:** Less than 25 years of ovarian reserve by assessment of AMH levels preoperatively and postoperatively.

Indicator/age ≤25	POG (n = 25)	RFG (n = 18)	p-value*
**AMH level (ng/ml)**			
Preop. AMH	2.67 (2.18–3.17)	2.80 (2.32–3.28)	0.717
In the first postop. m.	1.75 (1.35–2.15)^#^	2.78 (2.37–3.18)	0.001
In the third postop. m.	2.23 (1.78–2.67)^#^	2.70 (2.27–3.14)	0.132
In the sixth postop. m.	2.40 (1.94–2.86)	2.71 (2.28–3.14)	0.331

*As indicated by one-way ANOVA, ^#^As indicated by one-way ANOVA, the difference was statistically significant when compared with preoperative. AMH, anti-Müllerian hormone; POG, a postoperative observation group that underwent laparoscopic unilateral ovarian cystectomy; RFG, a referent group that did not undergo surgery; OARI, ovarian arterial resistance index; AFC, antral follicle count; Preop, preoperatively; in the first postop. m, in the first month postoperatively; in the third postop. m., in the third month postoperatively; in the sixth postop. m., in the sixth month postoperatively; (): The value is 95% confidence interval.

**Table 6:** More than 26 years of ovarian reserve by assessment of AMH levels preoperatively and postoperatively.

Indicator/age >26	POG (n = 31)	RFG (n = 42)	p-value*
**AMH level (ng/ml)**			
Preop. AMH	2.91 (2.47–3.35)	2.53 (2.17–2.88)	0.166
In the first postop. m.	1.53 (1.32–2.32)^#^	2.49 (2.01–2.87)	0.041
In the third postop. m.	2.01 (1.73–2.65)^#^	2.41 (1.94–2.67)	0.428
In the sixth postop. m.	2.66 (2.24–3.08)	2.55 (2.20–2.90)	0.679

*As indicated by one-way ANOVA, ^#^As indicated by one-way ANOVA, the difference was statistically significant when compared with preoperative. AMH, anti-Müllerian hormone; POG, a postoperative observation group that underwent laparoscopic unilateral ovarian cystectomy; RFG, a referent group that did not undergo surgery; OARI, ovarian arterial resistance index; AFC, antral follicle count; Preop, preoperatively; in the first postop. m, in the first month postoperatively; in the third postop. m., in the third month postoperatively; in the sixth postop. m., in the sixth month postoperatively; (): The value is 95% confidence interval.

**Figure 5:**
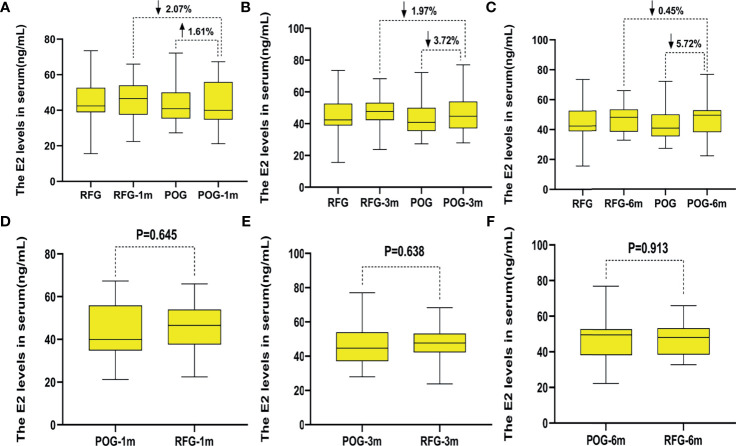
The changes of estradiol (E2) levels in the two groups. Compared with preoperative, the increased rate of E2 levels in postoperative observation group (POG) after the first postoperative month was 1.61%. Compared with E2 levels of referent group (RFG), the decline rate of E2 levels in POG after the first postoperative month was 2.07% **(A)**. Compared with preoperative, the decline rate of E2 levels in POG after third postoperative month was 3.72%. Compared with E2 levels of RFG, the decline rate of E2 levels in POG after the third postoperative month was 1.97% **(B)**. Compared with preoperative, the decline rate of E2 levels in POG after the sixth postoperative month was 5.72%. Compared with E2 levels of RFG, the decline rate of anti-Müllerian hormone (AMH) levels in POG after the third postoperative month was 0.45% **(C)**. The E2 levels showed no significant difference in POG in the first, third, and sixth month compared with RFG (p = 0.645, p = 0.638, p = 0.913, respectively) **(D–F)**.

**Figure 6:**
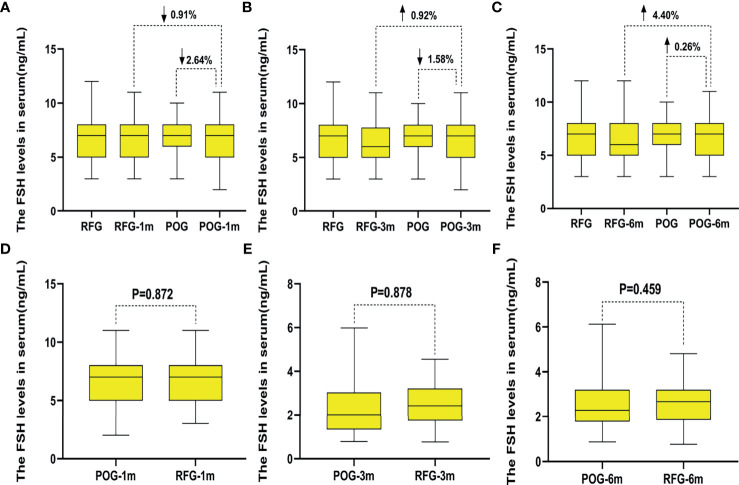
The changes of follicle-stimulating hormone (FSH) levels in the two groups. Compared with preoperative, the increased rate of FSH levels in postoperative observation group (POG) after the first postoperative month was 2.64%. Compared with E2 levels of referent group (RFG), the decline rate of FSH levels in POG after the first postoperative month was 0.91% **(A)**. Compared with preoperative, the decline rate of FSH levels in POG after third postoperative month was 1.58%. Compared with FSH levels of RFG, the increased rate of FSH levels in POG after the third postoperative month was 0.92% **(B)**. Compared with preoperative, the increased rate of FSH levels in POG after the sixth postoperative month was 0.26%. Compared with FSH levels of RFG, the increased rate of FSH levels in POG after the sixth postoperative month was 4.40% **(C)**. The FSH levels showed no significant difference in POG in the first, third, and sixth month when compared with that in RFG (p = 0.872, p = 0.878, p = 0.459, respectively) **(D–F)**.

The postoperative E2 and FSH levels were similar in the first, third, and sixth month between POG and RFG (p > 0.05; **Table 4** and **Figures 5**, **6**). There was no statistical significance between the three detection intervals (p > 0.05), between the detecting time point and treatment factors (p > 0.05), and between the treatment factors (p > 0.05).

## Discussion

In 2015, the American College of Obstetricians and Gynecologists recommended that ovarian reserve testing should be performed in all women who underwent ovarian surgery (22). Ovarian reserve testing could allow the individualization of treatment protocols to achieve optimal response while minimizing safety risks (16, 22). Therefore, we conducted this prospective cohort study to test the hypothesis that optimal time for recovery of ovarian reserve recovery after the laparoscopic unilateral ovarian non-endometriotic cystectomy occurred 6 months after surgery. The ovarian reserve decreased after the surgery. Also, in comparison with E2, FSH levels, OARI, and AFC, serum AMH levels could be used as a convenient and reliable marker for testing ovarian reserve in the short-term. Interestingly, AMH levels in POG showed a significant decline in the first postoperative surgery, which was 32.75% in POG and 26.67% in RFG; the AMH levels were more markedly reduced in POG than in RFG in the third and sixth postoperative month. The current study provided a clinical strategy for identifying the women with decreased or diminished ovarian reserve by detecting AMH every 6 months after laparoscopic unilateral ovarian non-endometriotic cystectomy. It is reasonable for the gynecologist and reproductive endocrinology physician to inform the women that their window of opportunity to conceive may be shorter compared to women of the same age who did not undergo laparoscopic unilateral ovarian non-endometriotic cystectomy, thus encouraging them to conceive sooner rather than later.

The best surrogate marker for oocyte quality is age (22). However, age can be defined as a rough indicator; thus, more useful and accurate indicators are needed for those younger women with decreased or diminished ovarian reserve. It is also reasonable to inform women that their fertile window may be shorter than anticipated before formulating an individualized treatment protocol. Over the years, various tests and markers of the ovarian reserve have been reported, such as the basal FSH in 1988, the AFC in 1997, and AMH in 2002 (23–25). The basal FSH plus E2 levels, AMH levels, and AFC should be used as appropriate ovarian reserve screening tests in clinical practice (22, 26).

The abnormally elevated FSH is almost equal with late DOR (high positive predictive value); yet, the majority of women being tested (including those with DOR) will show normal test results (low negative predictive value) (26). Therefore, a single FSH value has limited reliability (24). The high values of E2 have been associated with both poor ovarian response and failure to achieve pregnancy (27). The basal E2 has low predictive accuracy for poor ovarian response and failure to conceive; therefore, this test should not be used in isolation to assess ovarian reserve. Measurement of both FSH and E2 on cycle day 3 may help decrease the incidence of false-negative testing (28). In the current study, E2 and FSH levels were similar between POG and RFG in the first, third, and sixth postoperative month, respectively (p > 0.05); thus, E2 and FSH levels were not considered an effective and sensitive indicator of ovarian reserve changes in the short-term.

A previous study reported that AFC was correlated with the number of remaining follicles and that it had good intercycle and interobserver reliability (29). However, since AFC has inherent variability related to technology and interobserver variability (26), it is difficult to assess the exact number of antral follicles of the cystic ovary before cystectomy (30). According to existing literature, the OARI of patients with hypoestrogenic amenorrhea was decreased when compared with the eumenorrheic subjects (31). In the current study, AFC and OARI could not be used to evaluate ovarian reserve in the short-term, especially after laparoscopic ovarian non-endometriotic cystectomy.

AMH levels are increased in young adolescent women and peak at about 25 years of age, after which they gradually decline until reaching undetectable levels a few years prior to menopause (32). The level of AMH, which declines by 5.6% per year (33), reflects the size of the primordial oocyte pool (34). Testing with AMH has been reported at different times throughout the menstrual cycle (35). Recent studies have shown that serum AMH level is the most reliable and easily measurable marker for postoperative assessment of ovarian reserve, as it can show a postoperative decline (36, 37). It was reported that ovarian reserve evaluated with AMH was reduced in patients with ovarian endometriomas when compared with those with other benign ovarian cysts and with those with healthy ovaries (38), which was similar to our findings. The results indicated that the ovarian reserve decreased after the surgery and that, in comparison with E2 and FSH levels, OARI, and AFC, the serum AMH levels could be used as a convenient and reliable marker for testing ovarian reserve in the short-term. AMH levels showed a significant decline in POG when compared with those in RFG (1.88 *vs*. 2.57 ng/ml, p = 0.000) in the first postoperative month; the declining rate was significantly lower than the preoperative in POG (32.75%) as well as than that in RFG (26.67%). For the data of AMH levels stratified by age, the same trend was observed between less than 25 and more than 26 years old. AMH can be used to evaluate ovarian reserve after chemotherapy and gynecological operation. Nevertheless, some researchers have pointed out that AMH is not a hormone but a paracrine factor. The mechanism of AMH secretion into the peripheral circulation is unknown; however, there is sufficient evidence to suggest that the reduced serum AMH in women with endometriomas can indicate real and definitive damage to the ovarian reserve rather than only a transient and potentially reversible interference with ovarian physiology (39).

The mechanism of the decline of ovarian reserve following ovarian cystectomy remains largely unknown. This may be partially explained by the removal of some healthy ovarian tissue during laparoscopic ovarian non-endometriotic cystectomy, which contains a certain number of oocytes. It is also possible that the use of electrocoagulation for hemostasis in operation causes damage to the healthy ovarian tissue (5, 6, 40). A previous study showed that the number of acquired eggs was significantly decreased in *in vitro* fertilization (IVF) after surgery (41). Therefore, the hemostatic method in the current study was ensured with bipolar electrocoagulation forceps at the power of 25 W and for no more than 5 s, then with a loose knot made of 2/0 absorbable sutures for controlling bleeding. Thus, it is imperative that the ovarian tissue be protected as much as possible during the operation.

The current study has several limitations. The major one relates to the recruitment of patients with unilateral ovarian non-endometriotic cyst and the healthy women without ovarian cyst, excluding bilateral non-endometriotic cysts and endometrial cyst. Unilateral ovarian cysts could limit the impact on ovarian reserve, and the mechanism of the postoperative endocrine and paracrine changes in the healthy ovaries is still unknown. Consequently, AFC and OARI were detected at the preoperative and postoperative intervals, the AFC of cyst side of the POG group showed no difference in the first, third, and sixth postoperative month, and the OARI of cyst side of POG group showed no difference in the first, third, and sixth postoperative month. Therefore, the optimal time for recovery of ovarian reserve after the laparoscopic ovarian unilateral cystectomy is estimated to occur in the sixth month.

The postoperative measuring of the ovarian reserve was performed every 6 months for 2 years, depicting a change curve of ovarian reserve. We will continue our efforts to disentangle the intricate relations between ovarian cysts, ovarian reserve, and the impact of surgery. Future studies should aim to reveal the optimal interval for recovery of ovarian reserve for patients with unilateral ovarian non-endometriotic cyst and bilateral non-endometriotic cysts and endometrial cyst after surgery. Moreover, it is necessary to gain a better understanding of the mechanisms causing the decreased ovarian reserve to develop a clinical strategy and ameliorate the surgical techniques in use.

## Data Availability Statement

The original contributions presented in the study are included in the article/supplementary material. Further inquiries can be directed to the corresponding author.

## Ethics Statement

The studies involving human participants were reviewed and approved by Ethics approval (No. 2014031) was received from the Committee of Medical Ethics, Shanghai Punan Hospital. The patients/participants provided their written informed consent to participate in this study.

## Author Contributions

HL designed and performed the research, analyzed the data, and drafted the manuscript. BY analyzed the data and drafted the manuscript. ZL guided and provided the final approval of the manuscript. ZS, PL, YL, YiW, and XN collected and analyzed the data. All authors contributed to the article and approved the submitted version.

## Funding

The material purchases and the data collections in this project were supported by the Medical Leaders Training Program of the Health Bureau of Shanghai Pudong in China (No. PWRd 2016-15), Fund of Shanghai Pudong New Area Science and Technology Commission in Chin (No.PKJ2020-Y46).

## Conflict of Interest

The authors declare that the research was conducted in the absence of any commercial or financial relationships that could be construed as a potential conflict of interest.

## Publisher’s Note

All claims expressed in this article are solely those of the authors and do not necessarily represent those of their affiliated organizations, or those of the publisher, the editors and the reviewers. Any product that may be evaluated in this article, or claim that may be made by its manufacturer, is not guaranteed or endorsed by the publisher.

## Abbreviations

AFC, antral follicle count; AMH, anti-Müllerian hormone; FSH, follicle-stimulating hormone; OARI, ovarian arterial resistance index; POG, postoperative observation group; RFG, referent group.
